# The Billion Cell Construct: Will Three-Dimensional Printing Get Us There?

**DOI:** 10.1371/journal.pbio.1001882

**Published:** 2014-06-17

**Authors:** Jordan S. Miller

**Affiliations:** Department of Bioengineering, Rice University, Houston, Texas, United States of America

## Abstract

How structure relates to function—across spatial scales, from the single molecule to the whole organism—is a central theme in biology. Bioengineers, however, wrestle with the converse question: will function follow form? That is, we struggle to approximate the architecture of living tissues experimentally, hoping that the structure we create will lead to the function we desire. A new means to explore the relationship between form and function in living tissue has arrived with three-dimensional printing, but the technology is not without limitations.

## Abstract 2

Effective utilization of three-dimensional printing for tissue and organ engineering remains nontrivial. Here, Jordan Miller identifies key challenges and discusses conceptual targets on the horizon.

## Introduction

In the 1960s field known as Bionics, many human tissue functions were considered analogous to basic mechanical and electrical systems, such as servomechanisms [1]. Researchers made rapid progress recapitulating components of systems found in the body, and forecasts were made as to when human–machine interfaces would become so completely integrated with our anatomy as to be essentially undetectable. This conceptual framework has proven useful in practice, with contemporary work applied to human patients through surgical implants such as knee, hip, and limb prostheses [2]; pacemakers; and cochlear and retinal devices [3]. Although these medical devices significantly improve the quality of life for patients today, there are many functions in living tissues which cannot be addressed with electromechanical systems. Shrewd utilization of our best materials simply cannot replace tissues in the body whose functions are intimately tied to their biochemistry. For example, we don't know how to make a plastic or a metal that can metabolize acetaminophen and alcohol like the liver can.

Since cells are the major functional unit responsible for biochemistry in the body, efforts to separate cells from their native environment in vivo and apply them therapeutically in extracorporeal devices have remained steadfast. In extracorporeal liver-assist devices, live cells can be loaded into bioreactor chambers outside the body and then connected in a closed loop with host blood circulation so that the biochemical benefit from cells in the device will positively affect the patient [4],[5]. But these strategies that are external to the body, including dialysis of blood during kidney failure, lead to their own morbidities and are not suitable long-term therapies [6].

**Figure 1:**
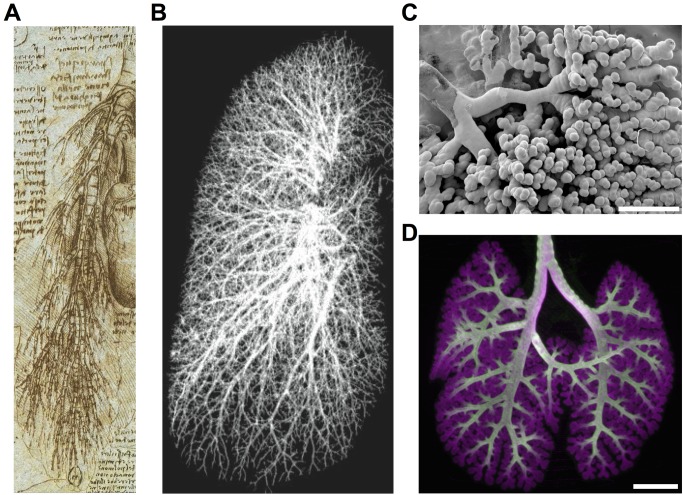
Anatomical complexity remains unsolved. (**A**) Leonardo da Vinci famously recognized the interpenetrating networks of lung vasculature and branched airways with his detailed drawings (c. 1500). Image courtesy of the European Union Leonardo Digitale. (**B**) Whole-lung vasculature can be reconstructed and visualized from computed tomography (CT) scans. Reprinted with permission from [61]. (**C**) Air sac architecture of adult rat lung (electron micrograph of decellularized resin cast). Image courtesy of Laura Niklason, additional research available via [25], scale bar  =  1 mm. (**D**) Optical projection tomography image of an embryonic day 15 mouse lung undergoing branching morphogenesis. Epithelium (E-Cadherin, magenta), future conducting airways (SOX2, white). Image courtesy of Jichao Chen, additional research available via [62], scale bar  =  500 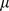
m.

Cells loaded into extracorporeal devices or growing at the bottom of a Petri dish bear little resemblance to the exquisite anatomical complexity found in the human body. Organs like the lung, heart, brain, kidney, and liver are pervaded by incredibly elegant yet frighteningly complex vascular networks (carrying air, lymph, blood, urine, and bile), leaving us without a clear path toward physical recapitulation of these tissues in the laboratory (Figure 1). However, we don't need to fully understand tissue organization or all of developmental biology (e.g., spatiotemporal growth factor release) before we can improve the quality of life for patients suffering from damaged or diseased organs. Transplanting whole organs from a human donor into a recipient can provide lifelong benefit when accompanied with immunosuppressive therapy [7],[8]. Moreover, isolated cells have been shown to be able to provide biochemical benefit to the host, even when injected or placed at ectopic sites inside the recipient [9]–[11].

As we look toward the future, the prospect of using a patient's own cells to develop living models of their active biochemistry as well as functional, life-lasting cellular implants offers potentially revolutionary changes to research and healthcare. Stem cell biologists are uncovering exciting new ways to induce pluripotency [12] and direct lineage commitment [13]. But simple questions about cell number and cell types, their spatial arrangement, and local extracellular and microenvironmental considerations remain largely intractable because of difficulties in placing and culturing cells in three-dimensional (3D) space. For example, embryoid body aggregates containing thousands of cells change differentiation trajectory as a function of cell population and microenvironmental characteristics [14], while larger cell populations packed at physiologic densities rapidly die because of lack of adequate oxygen and nutrient transport.

Recent advances in 3D printing, a suite of technologies originally developed for plastic and metal manufacturing, are now being adapted to operate within the soft, wet environments where cells function best. Because 3D printing excels at producing heterogeneous physical objects of high complexity, biologists and bioengineers are gaining unprecedented access to a rich landscape of tissue architecture we've always wanted to explore.

## Size Matters

Seminal work in the 1980s by Ioannis Yannas and colleagues [15] demonstrated that scar tissue formation in skin wounds could be blocked by a biocompatible, nontoxic implant made from a special formulation of collagen and glycosaminoglycans [16]. Notably, the implant further supported tissue regeneration such as normal collagen remodeling and ingrowth of functional nerves and blood vessels, yet remained devoid of more localized skin appendages such as hair follicles, sebaceous and sweat glands. Tissue engineering has since evolved to combine cells, a scaffold, and bioactive factors into a construct for study or implantation, with steady progress in the use of other conceptually simple and thin tissues such as cornea [17] and bladder [18], to restore function in human patients. Only one or two cell types are needed pre-implantation, and the body seems able to adequately make up the difference to get desired function.

**Figure 2:**
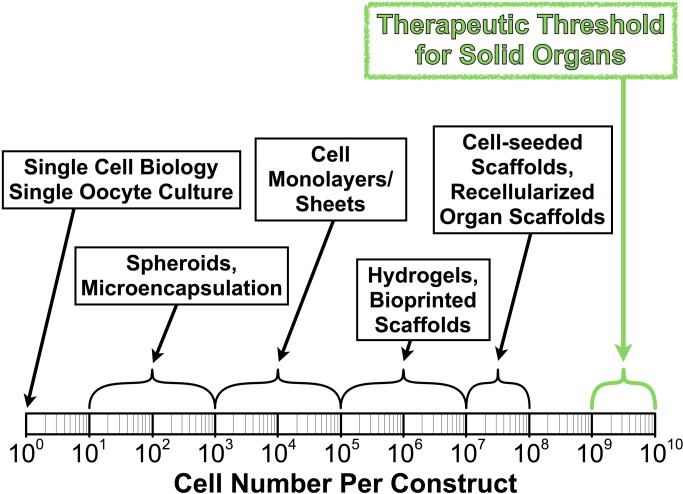
Tissue engineering. Investigations with engineered tissue constructs currently span at least eight orders of magnitude. Yet, the minimum therapeutic threshold for recapitulating solid organ function in humans is estimated at the level of 1–10 billion functioning parenchymal cells. We still have a ways to go.

Thin tissues require comparatively few cells. One study implanted a thin construct of hepatocytes subcutaneously in rodents and the cells were observed to proliferate and function normally [9], but such thin constructs cannot compensate for a whole liver. Scaling up tissue constructs is first and foremost a numbers game. Although humans are thousands of times larger than mice, human cells and mouse cells are about the same size. So, to translate thin tissue studies in mice to cellularized solid organ therapies for humans, we are going to need to be able to grow a lot more cells. With the minimum therapeutic threshold for solid organ replacement estimated at 1–10 billion functioning cells (Figure 2), current expertise in the field is still off by several orders of magnitude.

More subtly, the challenge is also one of cell density. More than 15 years ago, Laura Niklason and Bob Langer observed that "…gels seeded with cells have been limited by the fact that the resultant cell densities per unit volume that can be achieved are much lower than those observed in vivo…'' [19]. Little has changed here because of difficulties in keeping cells alive in culture at high density [20],[21]. Why does cell density matter? We know that many cells require close or direct contact with neighbors in order to function [22], whether during embryonic morphogenesis and development, homeostasis, or wound healing. So, just having 10 billion functioning cells growing across dozens of Petri dishes will not solve the problem. We will need to figure out how to organize cells into structures where their proper phenotype is reinforced.

Yet, it's not clear which anatomical components are necessary and sufficient for tissue function and which are superfluous trappings. Do we need microtissues with multiscale vasculature, or are organ-on-a-chip systems [23] adequate? Decellularized organs, which are then recellularized, have demonstrated great potential for tissue engineering applications [24]–[27] but lack a degree of architectural control which may be necessary for the experimentalist. To answer this question, we need tools which can dictate the cellular components, the extracellular matrix, and the interstitial fluidic space of engineered tissues with high precision in all three dimensions. Recent efforts in 3D printing are now providing exactly these capabilities, and automation and reproducibility are intrinsically built in.

## 3D Printing: Engineering Layer-by-Layer

Biologists and bioengineers are experts at adapting technologies developed in other industries for our own research endeavors. Techniques to organize or orient living cells on surfaces, for example, have come from modifying technologies from the microprocessor industry [28]. The arrival of 3D printing for the manufacture of objects of arbitrary complexity promises a sea change in tissue engineering and experimental biology.

**Figure 3:**
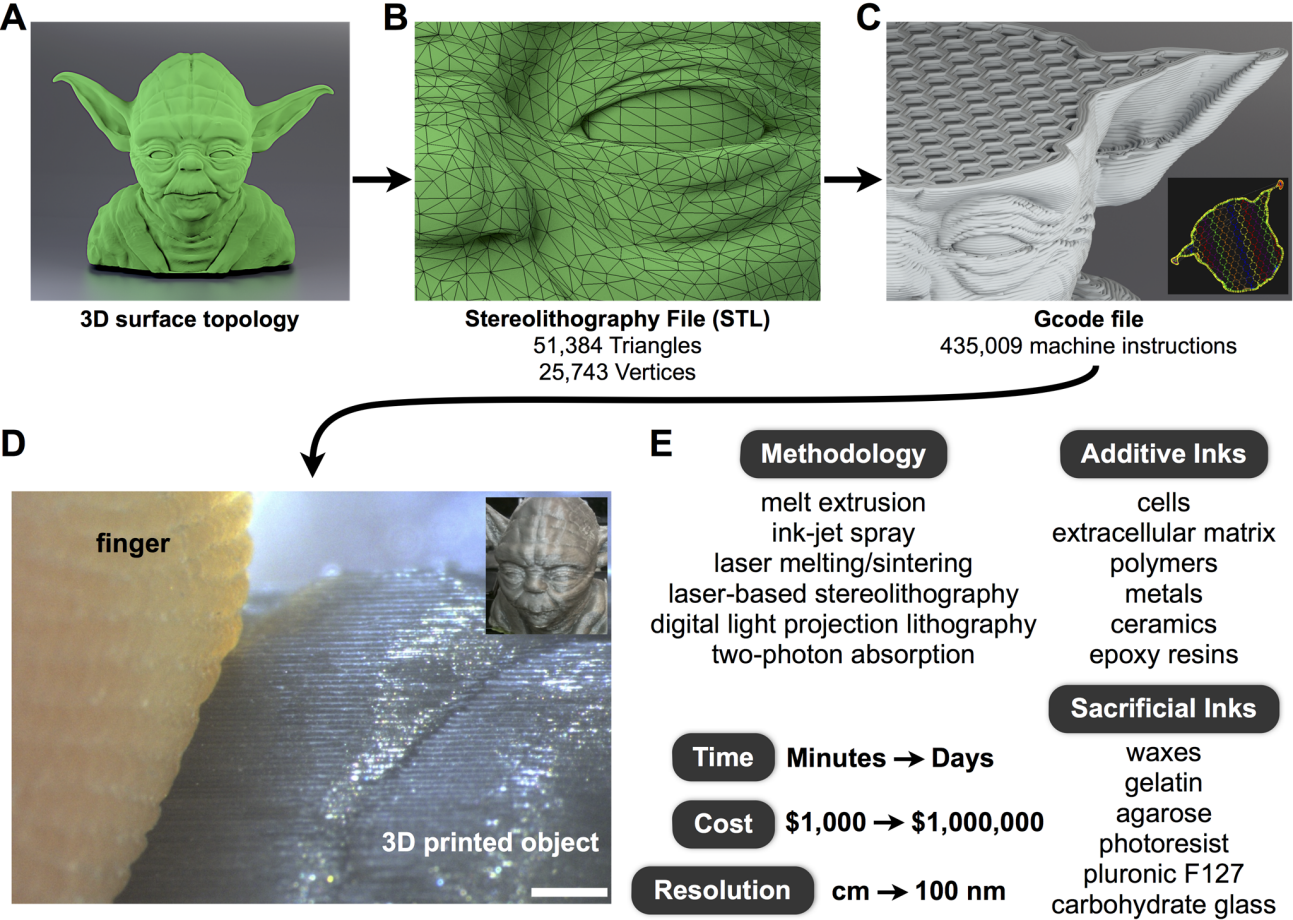
Overview of 3D printing. (**A**) A 3D model can be generated and visualized in a wide range of software packages. 3D model available under Creative Commons license via Thingiverse.com, courtesy of artists Barak Moshe and Faberdashery. (**B**) The surface topology is simplified to a mesh comprising a series of 3D coordinates (vertices) and the triangles (faces) that connect them. (**C**) The surface mesh is computationally sliced layer-by-layer to calculate machine instructions suitable for 3D printing. Machine instructions can be visualized en face or in cross-section (inset). (**D**) 3D printing via melt extrusion (inset) can easily achieve layer heights which surpass the resolution of human fingerprints. Scale bar  =  1 mm. (**E**) A selection of the diverse parameter space of 3D printing technologies. Many dozens of different combinations are in practice today.

3D printing is an iterative, additive technology. Rather than starting with a block of material and removing what is undesirable in a subtractive process (as in sculpting or milling), additive manufacturing starts from nothing and selectively builds, one layer at a time, an object of interest according to computer instructions. A dizzying array of technologies are currently in use, and all are potentially adaptable to engineering living tissues (Figure 3). An additive approach is unique among manufacturing technologies because it gives the user independent access to every (*x,y,z*) coordinate—termed "voxel'' (a portmanteau of volume and pixel)—within a given volume. Access to each voxel can make 3D printing rather slow: each time print resolution is doubled, the number of required voxels scales by a factor of eight (because 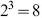). But the capabilities of 3D printing are best demonstrated in the fabrication of structures that cannot be made in any other way.

One conceptual approach to adapt 3D printing technologies for biology and medicine is a substitution of the commonly used resins (such as acrylates) and plastics (such as acrylonitrile butadiene styrene) with biocompatible, nontoxic materials like polylactic acid (PLA) and polycaprolactone (PCL). Recently, this approach was used to successfully treat an infant in respiratory distress. Doctors used noninvasive anatomical scanning to map the tracheal defect, then designed and printed a tracheal splint made entirely from PCL [29]. The patient achieved marked improvement in respiration, and the splint is expected to be fully resorbed within three years. Dental prostheses can be made with 3D printers in a doctor's private medical practice, further highlighting patient-specific customization advantages and broad clinical adoption of the technology [30].

To adapt 3D printing for the manufacture of living tissues, cells and extracellular matrix (ECM) are combined as ink in a process known as bioprinting. Inkjet printing [31], light-projection photolithography [32]–[34], and syringe-based extrusion [35],[36] allow the selective deposition of tissues in reproducible and heterogeneous patterns. Microvascular cells can be inkjet printed in fibrin [37], cellular aggregates made from chinese hamster ovary (CHO) cells or fibroblasts can be extruded among a supporting stroma of agarose [38], and primary aortic cells can be printed in the shape of their parent valve based on digitized microcomputed tomography (micro-CT) scans [39]. Besides ink considerations such as viscosity and crosslinking chemistry, cell handling is a major challenge. Some of the cell types most desirable for printing, such as hepatocytes, are actually quite fragile cells in culture; they may not survive the 3D printing process itself [31]. Taking inkjet printing as an example, although droplet ejection frequencies of around 20 kHz have been achieved [40], ejection of cells can induce transient nanopores in printed cells [41], which may explain some of the cell damage observed in this process. Just-in-time cell harvesting, microfluidic culture devices, or automated cell sheet manipulation [42] may improve the scalability and complexity of construct fabrication.

In contrast to these additive cellular inks, temporary inks can be printed, encased, and then selectively removed later [43],[44]. A distant relative of lost-wax casting, this sacrificial molding strategy trades most of the precision of specific cell placement for accurately structuring the negative space in tissues. For example, smooth channels and tubes can be patterned and perfused [45] to keep resident cells alive at densities not currently possible with bioprinting [21],[46]. The combination of sacrificial inks with bioprinting approaches may yield a hybrid strategy giving the best characteristics from each technique: heterogeneous cell patterning and perfusable vasculature [47].

Importantly, the ethos of the open-source software movement—making designs and code, like the Linux computer operating system, freely and legally available to anyone—has now bled into hardware designs and the software toolchain for 3D printers. The result is an explosion of more than 75,000 3D printers in operation worldwide by both researchers and hobbyists eager to help with focused scientific exploration [21],[48]. Yet, the most ubiquitous digital file format used for 3D printing—the stereolithography file (STL)—lacks any hierarchy to represent the structure of living tissue. The STL file only describes the surface of a 3D volume and contains no information about its internal space. The National Institutes of Health (NIH) has launched a new data bank for 3D printing (http://3dprint.nih.gov/) which may help to address this standardization problem. Protein crystallographers once wrestled with a similar challenge to define an all-inclusive file format for 3D data, resolved by the Research Collaboratory for Structural Bioinformatics (RCSB) open protein data bank (PDB) file format; nearly 100,000 protein structures have been centrally and publicly archived since 1971.

Simplification of the hardware and software tools required for 3D printing mean the technology is becoming accessible even to non-experts. The diversity of interests in 3D printing, coupled with the wide distribution of printers themselves, mean a plethora of opportunities exist for applying 3D printing to biology and medicine (Box 1).

**Box 1. Choose Your Own Adventure: An Abundance of Opportunities for 3D Printing**

**Cascading Signals**

The dynamism of biology is exemplified with spatial signaling cascades such as Notch, Wnt, and Hedgehog, and the ability to reconstruct models of their behavior in vitro is progressing steadily, with exquisite genetic control [63]. Applications of 3D printing to position specific cell types in three-dimensional arrangements and dictate their crosstalk may provide the experimental platform on which to test advanced multiscale computational models [64].

**Cooperativity and Morphogenesis**

To understand morphogenesis and cooperativity in large-scale tissues, we need techniques which can pattern cell populations to focus and understand their behavior [65],[66] in all three dimensions [67]. For example, models of angiogenesis, the sprouting of new blood vessels from pre-existing ones, are transitioning into 3D [46] and are backed by multiscale models [68]. When coupled with readouts that can measure cellular activity with spatiotemporal perspective, we might be able to better direct cellular motions and tissue deformations and stresses [69]–[71].

**Disease Progression**

Building physiologically relevant models of disease progression [72] is another area ripe for extension to 3D models. For example, in vitro models of tumor biology are providing new opportunities [69],[73]–[75]. In cancer, computational models of mass transport [76] have had difficulties making accurate predictions of chemotherapeutic potential because of the complexities of measuring, verifying, and correlating mass transport directly in patients; every tumor is heterogeneous and unique. Our changing understanding of cancer forces continued revision of conceptual models [77]–[79], which may benefit from 3D printing approaches, rigorous in vitro analysis [80], and correlation to human clinical data [81].

**Pharmaceutical Applications**

The promise of organ-on-a-chip systems [23] is that they physically model key aspects of human physiology with human cells. So it may be possible to bring high-throughput drug testing directly to cultured, vascularized human tissues fabricated with 3D printing. Further, since patient-specific responses to drugs are hard to predict, these technologies may one day make it possible to test drugs on cells cultured from specific patients, thereby helping to predict their best therapeutic cocktail and highest tolerable dose.

## Plugging It In

**Figure 4:**
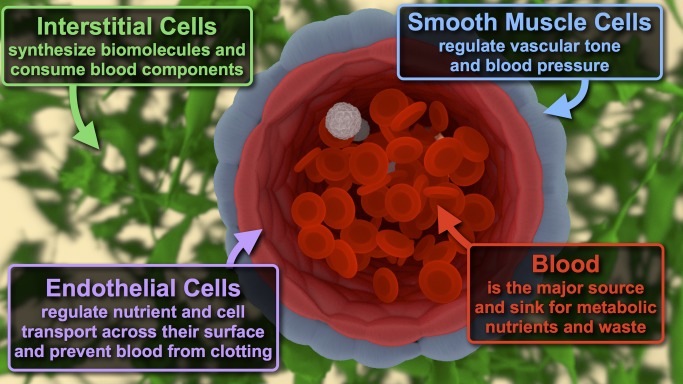
Journey of a molecular nutrient through native tissues. Cellular organization in vascularized tissues is commonly simplified into four regimes, which are rarely recapitulated together in engineered tissue constructs. Soluble blood components vary dramatically in size, concentration, and biochemistry, and each has distinct targets and mechanisms for negotiating tissue architecture. Artwork render and animation (Movie S1) performed with Blender.org open-source software.

The inexorable need for a continuous supply of oxygen and nutrients to maintain cell viability is a major limiting factor in the engineering of tissues containing living cells. Diffusion alone is sufficient for the growth of human cell aggregates up to several hundred micrometers thick; however, large cell aggregates develop necrotic cores. The challenge remains one of mass transport—how to get oxygen and nutrients in and waste products out of tissue constructs (Figure 4; animation available as supporting information Movie S1). Given the difficulties of mapping interstitial nutrient gradients in vivo, the path toward constructs containing billions of cells remains unclear.

Bulk perfusion of sponge-like macroporous tissue constructs in bioreactors can keep resident cells alive. However, seeded cells can secrete their own insoluble protein matrix into the porous void space, eventually restricting all mass transport [49]. Moreover, although parenchymal cells appear to be resilient to dramatic changes in their microenvironment, blood shows no such flexibility [50]. Perpetual difficulties in making simple extracorporeal devices and small-diameter vascular grafts (below 6 mm in diameter) have been hampered by fundamental hemodynamics and blood clotting biochemistry, especially in terms of acute and potentially lethal complications (e.g., stroke, heart attack, and pulmonary embolism). So, it's unclear how porous cellularized foams could be perfused with whole blood in the body.

To develop living tissue implants that can survive beyond the diffusion limits of oxygen in the interstitial fluids, we may need to construct new vascular networks that can be plumbed into the host vasculature and permit blood flow. Pre-vascularized implanted tissues can integrate into the host vasculature [51]–[53], but this takes anywhere from days to weeks. We will need to speed up the process for billion-cell constructs because necrosis can occur within hours, whether in engineered tissues or in donor organs for transplantation.

**Figure 5:**
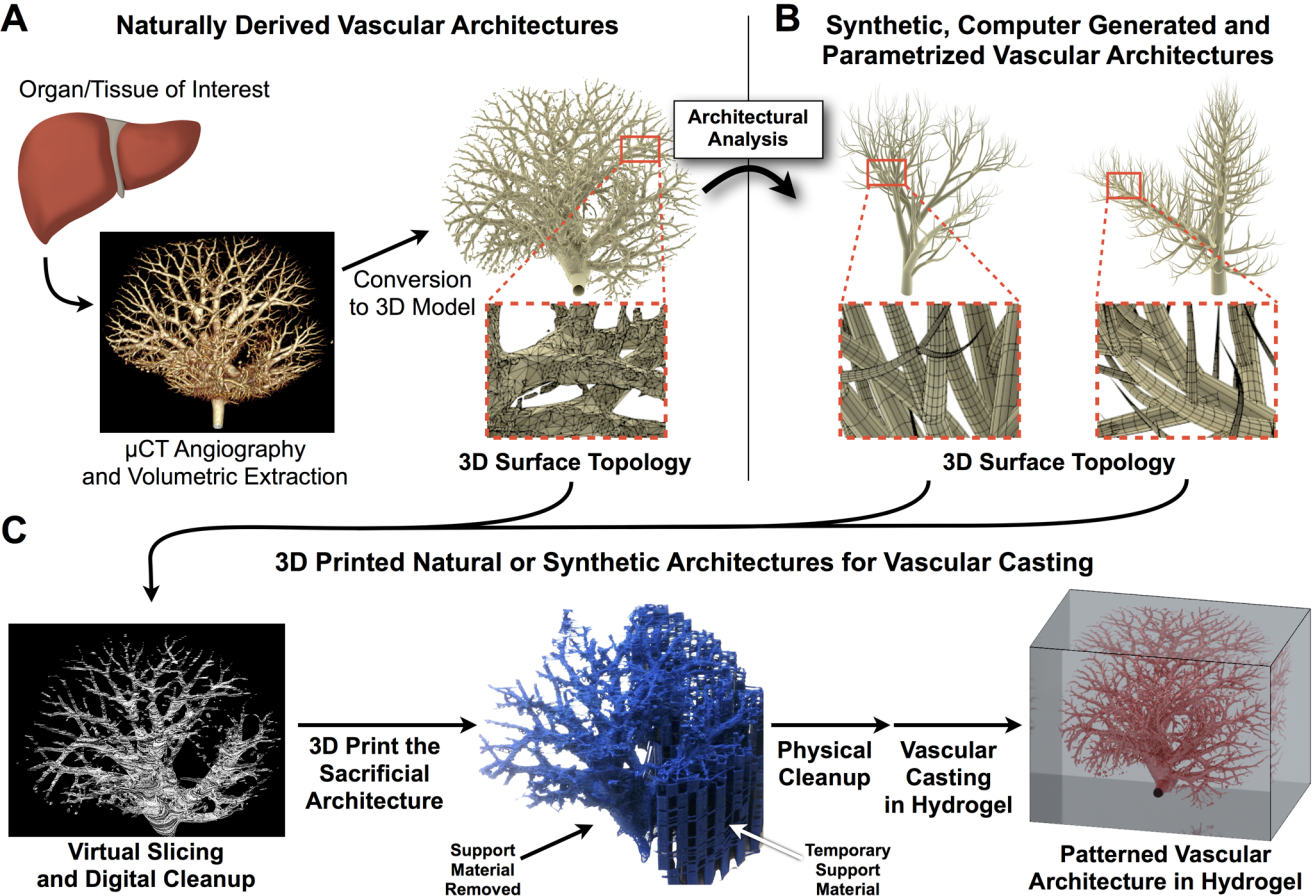
Recapitulating whole organ vasculature. It should be possible to create whole vascularized organoids by merging current anatomical mapping technologies with 3D printing. (**A**) A tissue or organ of interest is scanned via microcomputed tomography (micro-CT). Source 2D liver scans courtesy of Chris Chen and Sangeeta Bhatia, additional research available via [10]. The resulting voxels (volumetric pixels) can be visualized and converted into a 3D surface topology. (**B**) Optionally, the 3D surface mesh can be fully parametrized in order to generate, de novo, similar vascular architectures as a new topology. (**C**) Native or synthetically generated vascular architectures are then computationally sliced and prepared for 3D printing directly (in sacrificial ink) or by boolean volumetric subtraction (in additive ink). After physical cleanup, 3D printing can yield cell-laden hydrogels containing living cells and perfusable vasculature. Shown here for clarity is an architecture with one inlet and zero outlets, but more complete or complex architectures with multiple inlets and outlets could be achieved with this same workflow.

Basic anatomy demonstrates that identical organs from different people have unique vascular architectures, yet these organs can still function similarly for each person. While major arteries and veins are genetically encoded and form during embryogenesis [54]–[56], the microvasculature is remodeled based on local forces and needs [57]. Indeed, the vessel architecture of the retina is more distinct among people than their fingerprints. Thus, it is not necessarily the exact x, y, and z coordinates of individual vessels that permit organ function. Rather, the overall transport of blood components that results from vessel architecture is a principal factor defining healthy and diseased tissue (e.g., vessel tortuosity, red blood cell velocity, *pO*_2_, and pH). So, to solve transport questions in engineered tissues, it is likely that more than one architectural solution is possible (Figure 5).

Innervation of native vasculature is important for vasodilation and vasoconstriction and, thus, for regulating blood pressure throughout the body. But the neurovascular junction probably isn't critical for initial attempts to synthesize living tissue. Transplanted human hearts, for example, are not surgically tied to the nerves of the recipient; they beat at their own pace. And proximal sensory nerves can innervate and restore feeling in regenerated skin substitutes. So it may be that such re-innervation will work in other engineered organ systems. Individual neuronal processes running up to several feet in length will not likely be deposited with 3D printing anytime soon, although the concept of 3D printing inside shear-thinning gels [45],[47] provides an intriguing opportunity for neural printing, because long strands are easily deposited.

## Building the Future

Once questions of architecture for a given tissue construct are answered in the laboratory, extending toward human therapy will require addressing a new set of challenges. Constructs made by 3D printing, especially those containing living cells, are subject to an evolving regulatory pathway to the clinic for treatment of human patients. Recent reviews shed light on some of these hurdles, such as how to keep tissue fabrication sterile, quality assurance, and the changing landscape of venture funding for human clinical trials [58],[59]. Designing 3D printing systems with these good manufacturing practice (GMP) considerations already planned or incorporated can only benefit the translational workflow from research to development, albeit at significantly increased cost. In particular, there are immediate opportunities for developers of biomaterial inks and 3D printers to commercialize their work for use in experimental research.

We are still at the early stages, with access to 3D printing technologies expanding at rates akin to the personal computer revolution of the 1980s. Standardization and automation of tissue assembly, especially when based on open-source or publicly disclosed standards, will continue to aid in reproducibility across laboratory groups, just as polystyrene Petri dishes have standardized monolayer cell culture. Besides new fabrication technologies, we also need better metrics for measuring engineered tissue function. The size and cell density of engineered tissues is now approaching that of a mouse itself. Consequently, non-invasive imaging and other related methodologies developed primarily for assessing animal models [57],[60] will be applied to tissue engineering research questions with increased attention. So how many different vascular networks will we need to build into engineered tissues for biology and medicine? The answer is that we simply don't know, so let's start with one.

## Supporting Information

Movie S1 **Journey of a molecular nutrient through native tissues.** Cellular organization in vascularized tissues is commonly simplified into four regimes, which are rarely recapitulated together in engineered tissue constructs. Soluble blood components vary dramatically in size, concentration, and biochemistry, and each has distinct targets and mechanisms for negotiating tissue architecture. Artwork render and animation performed with Blender.org open-source software.
(MP4)
